# Effect of Prostanoids on Human Platelet Function: An Overview

**DOI:** 10.3390/ijms21239020

**Published:** 2020-11-27

**Authors:** Steffen Braune, Jan-Heiner Küpper, Friedrich Jung

**Affiliations:** Institute of Biotechnology, Molecular Cell Biology, Brandenburg University of Technology, 01968 Senftenberg, Germany; steffen.braune@b-tu.de (S.B.); Jan-Heiner.Kuepper@b-tu.de (J.-H.K.)

**Keywords:** prostacyclin, thromboxane, prostaglandin, platelets

## Abstract

Prostanoids are bioactive lipid mediators and take part in many physiological and pathophysiological processes in practically every organ, tissue and cell, including the vascular, renal, gastrointestinal and reproductive systems. In this review, we focus on their influence on platelets, which are key elements in thrombosis and hemostasis. The function of platelets is influenced by mediators in the blood and the vascular wall. Activated platelets aggregate and release bioactive substances, thereby activating further neighbored platelets, which finally can lead to the formation of thrombi. Prostanoids regulate the function of blood platelets by both activating or inhibiting and so are involved in hemostasis. Each prostanoid has a unique activity profile and, thus, a specific profile of action. This article reviews the effects of the following prostanoids: prostaglandin-D_2_ (PGD_2_), prostaglandin-E_1_, -E_2_ and E_3_ (PGE_1_, PGE_2_, PGE_3_), prostaglandin F_2α_ (PGF_2α_), prostacyclin (PGI_2_) and thromboxane-A_2_ (TXA_2_) on platelet activation and aggregation via their respective receptors.

## 1. Introduction

**Figure 1:**
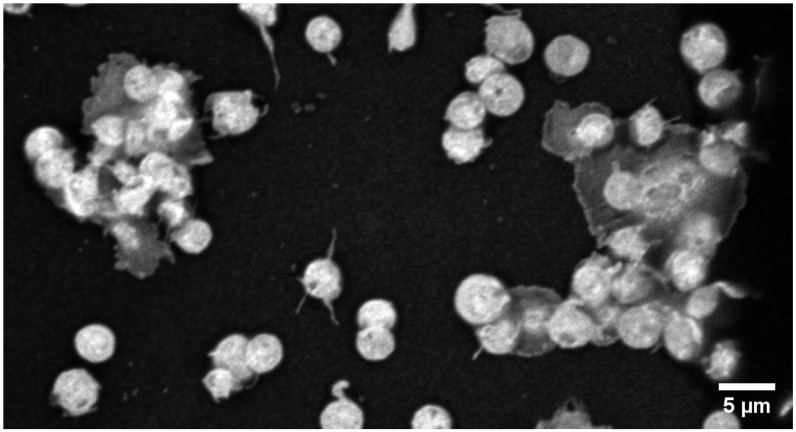
Morphology of activated platelets and platelet-aggregates adherent on collagen after 60 min treatment with platelet rich plasma. Adherent platelets were treated with a 2% glutardialdehyde solution for visualizing the platelet body unspecifically (Glutardialdehyde Induced Fluorescence Staining). Microscopy was conducted at 100-fold primary magnification with a ZEISS LSM800 in the high resolution AIRYSCAN-mode.

Hemostasis is a complex process that requires the interplay of multiple physiological pathways. Cellular and molecular mechanisms interact to stop bleedings of injured blood vessels or to seal denuded sub-endothelium with localized clot formation (Figure 1). Once vascular integrity is restored, clot formation stops and normal hemostasis is reinstated. Thrombotic imbalance may occur in patients with atherosclerotic diseases and activated platelets. The latter expose a plethora of receptors (e.g., CD62P and PAC1) and phosphatidylserine on their plasma membrane, resulting in the recruitment of circulating platelets (thrombus formation) as well as the binding and activation of the prothrombinase complex (thrombin formation) [1]. Activated platelets further mediate thrombotic processes and hemostasis by releasing bioactive substances such as growth factors, chemokines, Ca^2+^, adenosine diphosphate (ADP/ATP) as well as phospholipids [2,3]. Accordingly, hyperreactive platelets play a critical role in several pathological conditions such as atherosclerosis [4,5,6], stroke or myocardial infarction [7,8,9,10], but also after the implantation of cardiovascular implants [11,12,13]. Despite the successful application of anti-platelet therapies, it remains challenging to sufficiently impair the hyperreactivity of platelets, while balancing medication-induced risks for major bleedings. Here, we review the present literature data available on the influence of prostanoids on platelet function and their therapeutic potential in cardiovascular diseases.

## 2. Generation of Prostanoids in Platelets

**Figure 2:**
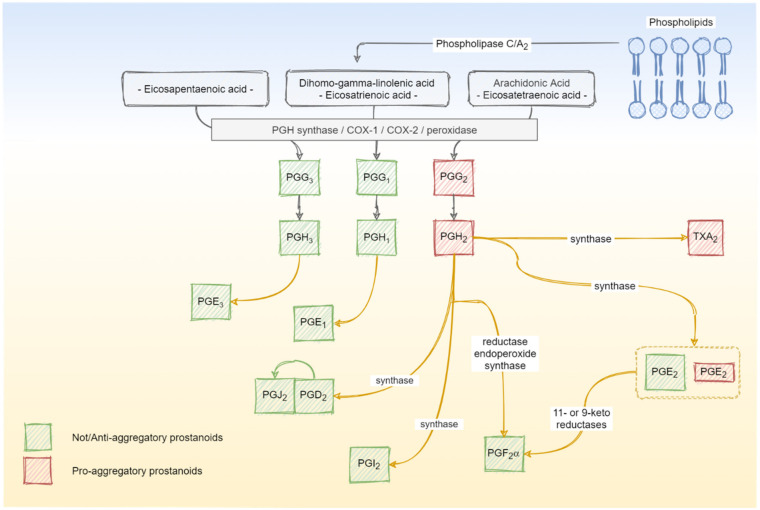
Overview of the major sources and biosynthesis routes of pro- and anti-platelet aggregatory prostanoids in the dense tubular system of human platelets.

Following the primary activation by, e.g., collagen and thrombin, bioactive lipids are formed in the platelet, which support consolidation of the activation process [14]. Most of these substances originate from free fatty acids such as arachidonic acid, the most common fatty acid in the platelet phospholipid membrane (Figure 2). Prostanoids are a family of these lipid mediators and consist of prostaglandins, prostacyclins and thromboxanes. The prostanoids are not stored in a reservoir but are synthesized de novo and released into the extracellular space when platelets are activated and exogenous free arachidonate is supplied [15]. The major site for prostanoid biosynthesis in the human platelet is the dense tubular system (DTS) [16,17]. This endomembrane system forms a residual smooth endoplasmatic reticulum (ER) and originates from the rough ER of the platelet shedding megakaryocytes. The elongated and irregularly formed organelle is located near the plasma membrane and microtubules. The DTS stores calcium as well as thromboxane synthetase, prostaglandin G/H synthase and cyclooxygenase (COX) [18]. These enzymes can transfer C-20 polyunsaturated fatty acids—mainly dihomo-gamma-linoleic (20:3n-6), arachidonic (20:4n-6), and eicosapentaenoic (20:5n-3) acids—into their oxidized active form, which are then released into the extra-platelet space.

Particularly, COX-1 is the dominant—but not exclusive—source of prostanoids in platelets. COX-2 is located in the vasculature induced by cytokines or shear stress and is the more important source of prostanoid formation in inflammation. However, both enzymes contribute to the generation of autoregulatory and homeostatic prostanoids. Five primary prostanoids are described today: prostaglandin-D_2_ (PGD_2_), prostaglandin-E_1_ (PGE_1_), prostaglandin-F_2α_ (PGF_2α_), prostacyclin (prostaglandin-I_2_), and thromboxane-A_2_ (TXA_2_). Each of them signals through a distinct transmembrane guanosine-5’-triphosphate-(GTP) binding protein coupled receptor.

## 3. Prostanoid Receptors

Prostaglandins and thromboxane bind to cognate receptors: Prostaglandin-D_2_ receptor (DP_1_), Prostaglandin-E_2_ receptor (EP), Prostaglandin-F_2_ receptor (FP), Prostaglandin-I_2_ receptor (IP) and TXA_2_ receptor (TP) [19,20]. There are four subtypes of prostaglandin-E_2_ receptors: EP1, EP2, EP3 and EP4 [21]. In these four subtypes, EP3 is unique and has several isoforms derived from alternative splicing [22,23].

In addition to these eight types and subtypes, a further receptor for prostaglandin-D_2_ exists: the chemoattractant receptor-homologous molecule expressed on T-helper type 2 cells (DP_2_, CRTH_2_). However, it has no significant sequence homology of amino acids with the prostaglandin-D_2_ receptor DP_1_ and other prostanoid receptors [24]. Via these receptors, prostanoids exert a variety of actions in various tissues and cells [25]. The regulation of platelet function is one of their most studied actions [26,27].

Several prostanoid receptors are expressed in human platelets: DP_1_ along with EP2, EP3, EP4, IP and TP [28,29]. Five with six subtypes are established so far. Table 1 summarizes these receptors, their G-protein and the respective signaling pathways. The following receptors regulate adenylyl cyclase (AC): IP, DP1, EP2, EP3, EP4, TP, DP2. While inhibition of AC results in a decrease in cyclic adenosine monophosphate (cAMP), its activation leads to an increase of this secondary messenger [30,31]. Further second messenger molecules are formed upon activation of phosphatidylinositol _3_-kinase (PI_3_K) by EP4: PI _3_-phosphate, PI _(3,4)_-bisphosphate, and PI _(3,4,5)_-trisphosphate [32]. Activated phospholipase-C (PLC) induces the generation of diacylglycerol (DAG) and inositol trisphosphate (IP3) secondary messengers [33]. Both are responsible for raising cytosolic Ca^2+^ levels and, thus, calcium-dependent pathways of platelet activation. Also, protein kinase-C (PKC) secondary messengers are activated through IP3 and DAG formation. Activation of p38 mitogen-activated protein kinases (p38 MAPK), the extracellular signal-regulated kinases (ERK), as well as the cAMP-response element-binding protein (CREB) leads to phosphorylation (influence activity) of key proteins that govern platelet function [34,35].

In the following paragraphs, an overview about stimulatory and inhibitory influences of prostanoids on platelet activation are depicted.

### 3.1. Thromboxane A_2_ (TXA_2_)

The main prostanoid produced by activated platelets and endothelial cells is TXA_2_. Beyond its generation in platelets, it is also released by endothelial cells and has prothrombotic properties [36,37,38,39]. The prothrombotic molecule is very unstable in aqueous solutions since it is hydrolyzed within about 30 s to the biologically inactive thromboxane-B_2_ (TXB_2_, half-life time 5–7 min, plasma levels: 2 to 285 pg/mL) [40,41,42,43]. Due to its short half-life, it primarily functions as an autocrine or paracrine mediator in the tissues adjacent to its site of generation. Beyond its influence on platelets, it acts as a vasoconstrictive, and mediates angiogenesis and inflammatory processes [44].

**Figure 3:**
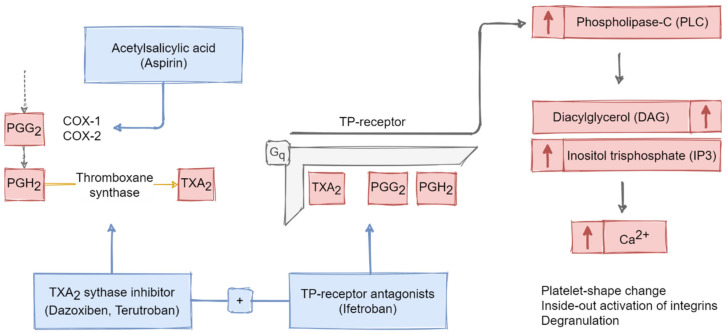
Schematic overview of the molecular pathways responsible for TXA_2_- as well as its precursors PGG_2_- and PGH_2_-mediated induction of platelet activation via the TP-receptor. Activating processes comprise downregulation of AC and upregulation of PLC. These processes lead to the elevation of calcium mobilization and secretion through DAG and IP3 activation. Therapeutic options include blockage of the COX-mediated synthesis of TXA_2_ as well as the combined administration of TP-receptor antagonists and TXA_2_ synthase inhibitors (red arrows in boxes pointing upwards indicate activation or increase of the respective substance).

TXA_2_ binds to the TP_a_ receptor, which results in TXA_2_-induced platelet-shape change, inside-out activation of integrins, and degranulation (Figure 3) [45]. The receptor couples to the PLC stimulatory G-protein (G_q_) and activates it. This leads to the elevation of intracellular Ca^2+^ concentrations, released from the DTS.

In human platelets, a stable TXA_2_ mimetic induced platelet aggregation and the release of granule contents from platelets [46]. This was followed by an amplification loop, which led to further platelet activation, aggregation and TXA_2_ formation [47]. Platelets express the TP receptor constitutively and generate TXA_2_ when activated with collagen, adenosine diphosphate (ADP), epinephrine, thrombin or TXA_2_ itself. Whereas elevated levels of TXA_2_ are associated with thrombotic and ischemic events, deficiencies can result in bleeding [48,49,50]. Thus, TXA_2_ plays an important role as a positive feedback regulator in the regulation of platelet function.

Therapeutically, acetylsalicylic acid (Aspirin) is classically applied to reduce the risk for acute coronary events through inhibition of the COX-mediated generation of TXA_2_ and prostaglandin endoperoxides.

Beyond its primary agonist TXA_2_, the TP receptor is also available for its metabolic precursors prostaglandin G_2_ (PGG_2_) and prostaglandin H_2_ (PGH_2_) [51]. Binding of both to TP showed similar platelet responses compared to TXA_2_. These findings became particularly important for the clinical application of TXA_2_ synthase inhibitors (e.g., Dazoxiben). Despite the fact that the metabolites did reduce TXA_2_ production and stimulated the generation of anti-aggregatory PGE_2_, PGD_2_, PGI_2_ and PGF_2α_, they led to an accumulation of the pro-aggregatory precursors mentioned above [52,53].

Limitations of this approach could be reduced through the combined administration of TXA_2_ synthase inhibitors and TP receptor antagonists (e.g., Terutroban and Ifetroban) [54,55]. The latter were shown to reduce TXA_2_­ (or precursors)-induced platelet aggregation and shape change in patients in a comparable manner to Aspirin [56,57].

### 3.2. Prostaglandin-E_2_ (PGE_2_, Low Concentrations)

Prostaglandin-E_2_ is a lipid, arachidonic acid-derived, prostaglandin hormone. It is a product of the arachidonic acid metabolism in varying cells, including smooth muscle cells, colon cells, fibroblasts, platelets and macrophages, and plays an important role in inflammation as well as cancer [58,59,60].

In the human microvasculature, PGE_2_ is the main prostanoid secreted by endothelial cells [61] and can influence the vascular tone and angiogenesis [62]. In atherosclerotic plaques, activated macrophages contribute to elevation of PGE_2_ levels, which triggers platelet activation during plaque growth and upon rupture [63,64].

In vivo, PGE_2_ is rapidly converted to an inactive metabolite (13,14-dihydro-15-keto prostaglandin-E_2_) by the prostaglandin 15-dehydrogenase pathway. Its half-life in the circulatory system is approximately 30 s. Normal plasma levels range between 3 and 12 pg/mL [65].

**Figure 4:**
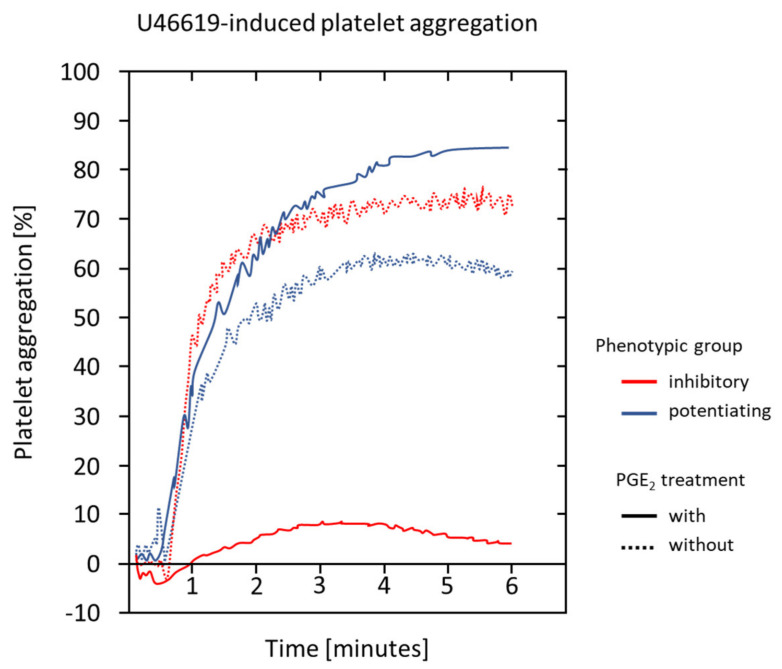
Representative light transmission platelet aggregation (LTA) curves showing the phenotypic differences in the response to low dosages of PGE_2_ (100 nmol/L, 30 s PGE_2_ treatment prior activation with submaximal concentration of U46619, LTA with adjusted platelet-rich plasma with 250,000 platelets per µL). Adapted from Friedman and colleagues [44].

Prostaglandin-E_2_ has been reported to have a biphasic effect on platelet activation. It potentiates, e.g., the U46619-iduced platelet aggregation, at lower concentrations (e.g., 0.1–10 μmol/L) and inhibits it at higher concentrations (e.g., >10 μmol/L) (see Figure 4) [66,67,68,69]. However, alone, it is not sufficient to induce platelet aggregation as a consequence of the strong counteracting AC stimulation of other prostanoids [70].

**Figure 5:**
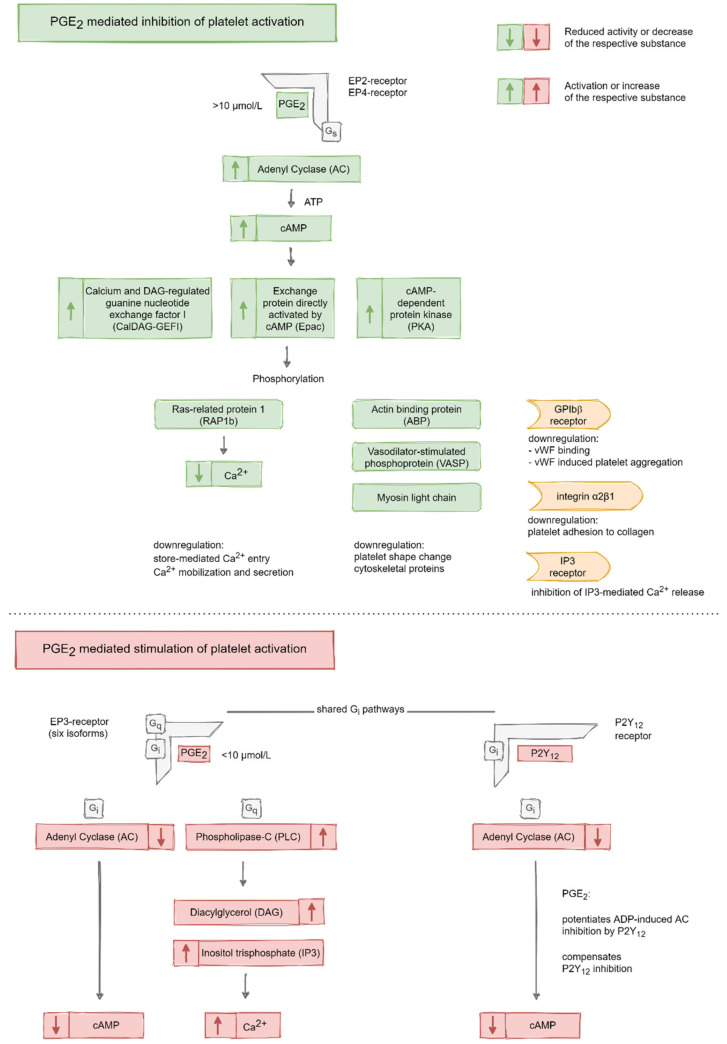
Schematic overview of the molecular pathways responsible for PGE_2_-mediated inhibition (PGE_2_ concentration > 10 µmol/L, via EP2- and EP4-receptors) and stimulation (PGE_2_ concentration < 10 µmol/L, via EP3- receptor) of platelet activation. The inhibitory pathways comprise activation of AC and increase of cAMP. This induces CalDAG-GEFI, EPAC and PKA signaling, which results in the phosphorylation of different proteins as well as (plasma membrane) receptors and the downregulation of calcium mobilization. Processes inducing platelet activation comprise downregulation of AC and upregulation of PLC. These processes result in the reduction of cAMP and in an elevated calcium mobilization and secretion through DAG and IP3 activation. The EP3- and P2Y_12_-receptors share the G_i_ pathway. Through this common pathway, PGE_2_ can potentiate the P2Y_12_-receptor-mediated and ADP-induced AC inhibition and, furthermore, compensate P2Y_12_ inhibition to a certain extent.

It has been thought that platelet activation can be induced by cAMP inhibitory (G_i_) and PLC stimulatory G-protein (G_q_) signaling (EP3 receptor). This is counteracted by induction of the cAMP stimulatory G-protein (G_s_) pathway, which can inhibit platelet activation (EP4 and EP2 receptors) (Figure 5) [63,69,71,72,73]. By coupling to G_i_, EP3 causes an inhibition of the AC. This leads to the above-mentioned decrease in the intra-platelet cAMP concentration and thus reduces the platelet activation threshold [63,72,74,75]. EP3 shares the G_i_ protein pathway with the ADP-dependent P2Y_12_ receptor. Through this, PGE_2_ can potentiate the ADP-induced AC inhibition by P2Y_12_ and—to some extent—even compensate P2Y_12_ inhibition by, e.g., pharmacological antagonists [76]. Beyond this classical view, the EP3 receptor appears more complex. Six isoforms are described, which elevate cAMP and IP3 levels through G_s_, G_q_ and G_z_ binding, differently [77,78,79,80,81].

The receptor subtypes EP4 and EP2 (both G_s_-coupled) are regarded as inhibitory receptors, which induce AC and thus cAMP generation from ATP [82]. Elevated cAMP levels can target different pathways but majorly the cAMP-dependent protein kinase (PKA) pathway [83]. Through the binding of cAMP to the regulatory subunits of PKA, its catalytic subunits become activated and can phosphorylate several substrate proteins responsible for inhibiting platelet activation. These cAMP-dependent processes include induction of the exchange protein activated by cAMP (Epac) as well as calcium and DAG-regulated guanine nucleotide exchange factor I (CalDAG-GEFI) and, thus, Rap1 signaling [84,85,86,87,88,89]. The cAMP-mediated phosphorylation of Rap1b leads to its dissociation from the sarcoendoplasmic reticulum Ca^2+^-ATPases 3b (SERCA 3b), which stimulates SERCA 3b activity to fill the associated calcium pools in platelets [90]. This was shown to inhibit calcium mobilization and thus platelet aggregation [91,92].

Data about the recruitment of arrestins by PGE_2_-activated EP4, indicated that—in HEK 293 cells—EP4 signaling may also comprise cAMP-independent pathways [93]. Furthermore, anti-inflammatory signaling was shown for the EP4 receptor-associated protein (EPRAP) in human macrophages [94]. In genetically engineered HEK-293 cells, EP4 signaling has been reported to activate PI_3_K, leading to activation of protein kinase-B (PKB/AKT), extracellular signal-regulated kinases (ERK), as well as cAMP-independent recruitment of arrestins (PAR4-dependent pathway) [95,96]. However, it remains to be confirmed that these data are transferable to human platelets.

Phenotypic differences in the response of human platelets to low concentrations of PGE_2_ (e.g., 0.01–5 and 100 nmol/L) have been shown, particularly in studies concerning the development of thromboxane synthase inhibitors [71,97,98]. In apparently healthy subjects, two groups were characterized showing inhibitory (45%, also termed responders) and potentiating effects (55%, also termed non-responders) of PGE_2_ on platelet aggregation (see Figure 2) [71]. Two mechanisms have been suggested, which may explain this variability: (1) subject-dependent variations in the PGE_2_ + TXA_2_ to PGD_2_ ratio and (2) in the responses of the AC to PGE_2_ [99]. However, these interindividual differences diminished when platelets were treated with high concentrations of fully activating antagonists [71].

### 3.3. Prostaglandin-F_2α_ (PGF_2α_)

Watanabe et al. described the formation of PGF_2α_ majorly as a result of the PGH_2_ reduction by prostaglandin reductase and endoperoxide synthase. The generation of PGF_2α_ through the conversion of PGD_2_ and PGE_2_ by 11- or 9-keto reductases was shown as well [100]. The half-life time of PGF_2α_ is less than one minute, after which it is enzymatically degraded into the more stable 15-keto-dihydro-PGF_2α_ [101]. PGF_2α_ is present in most of the human tissues and majorly abundant in the reproductive system of females [102,103]. In different mice tissues but also in human endometrial adenocarcinoma (Ishikawa) cells, this prostanoid binds to the FP receptor, which couples to the G_q_ [104,105,106]. Activation of FP by PGF_2α_ results in the IP3 and DAG formation as well as in the mobilization of Ca^2+^ [106,107,108].

Zhang et al. have provided a substantial overview of the actions of PGF_2α_ in different tissue cells and species [109]. Here, we want to focus on the function of PGF_2α_ signaling in platelets. It is noteworthy that, in the cardiovascular system, the prostanoid is mainly generated by fibroblasts in the cardiac tissue where it can induce arrhythmia, hypertrophy and fibrosis [110]. Increased levels were shown in the canine endocardium after induced cardiac ischemia and reperfusion [111]. Also increased levels of PGF_2α_ secretion were reported for vascular endothelial cells upon shear stress exposure [112]. In vascular smooth muscle cells, PGF_2α_ can induce resistance artery constriction [110,113,114].

The early studies of Hung, Armstrong, and coworkers have shown that PGF_2α_ (and the 8-epi-metabolite, 8–15 µM) can antagonize platelet aggregation induced by TXA_2_ (human platelets), PAF and thrombin [115,116]. Interestingly, in human platelets, ADP-induced aggregation was not affected, while in murine platelets, it was enhanced in a concentration-dependent manner [116,117]. The sole administration of PGF_2α_ had no effect on platelet activation. Receptor blocking experiments in mice revealed that PGF_2α_ can decrease cAMP levels via the EP3 receptor and increase IP3 levels (and Ca^2+^) through the interaction with the TP receptor [117]. In murine platelets, interaction of PGF_2α_ with the FP receptor could not be confirmed. The partly contradictory results concerning the actions of PGF_2α_ in human platelets underline the necessity of further studies on this prostanoid.

Synthetic derivatives of PGF_2α_ (Latanoprostene) are used, e.g., in ophthalmology to reduce intraocular pressure [118]. A clinical application as a platelet inhibitor is not in use.

### 3.4. Inhibitory Effects of Prostanoids on Platelet Aggregation

#### Prostaglandin-I_2_ (PGI_2_, Prostacyclin)

Prostaglandin-I_2_ was firstly described by Moncada et al. in 1976 and is majorly synthesized by endothelial cells and smooth muscle cells [27,119]. It is metabolized rapidly, and has a very short half-life time of about 42 s in humans [120], after which it is inactivated (non-enzymatically) and forms 6-ketoprostaglandin-F_1α_ [121]. Counteracting the prothrombotic properties of the platelet-derived TXA_2_, the endothelial PGI_2_ efficiently inhibits platelet activation, particularly in healthy blood vessels and under elevated shear flow [27,122,123,124]. Its inhibitory potential is higher than that of the other inhibitory prostanoids such as PGD_2_ and PGE_1_ [125]. PGI_2_ binding to the associated IP receptor (coupled to G_s_) leads to an activation of the AC and thus to an increase of intracellular cAMP. Its elevation downregulates store-mediated calcium entry, calcium mobilization and secretion, as well as platelet adhesion to subendothelial collagen via integrin α2β1 [31,126,127,128]. The cAMP increase further results in an activation of protein kinase-A (PKA) and in principle, in an inhibition of platelet activation. Analogous to cAMP, PKA activity has been associated with a reduced Ca^2+^ release from intra-platelet stores [129,130]. However, several other substrates of PKA and respective pathways have been described. Its actions include the regulation of platelet shape change and cytoskeletal proteins, e.g., through phosphorylation of the actin binding protein (ABP) and vasodilator-stimulated phosphoprotein (VASP), or through inhibition of myosin light-chain phosphorylation [131]. Activated PKA also phosphorylates receptors such as GPIbβ—a subunit of the VWF-binding GPIb-IX complex [132]—and the IP3 receptors on the DTS [130,133]. Furthermore, PGI_2_ has a vasodilatory effect, which increases blood flow, particularly in the microvasculature. In addition, PGI_2_ can also exert long-term effects such as promoting angiogenesis [134], primarily through the receptors IP and EP4 [135].

A study by Smith and Silver revealed that bleeding time in mice lacking this receptor was not different from that in wild-type mice. However, the susceptibility of the receptor-deficient mice to establish thrombosis was increased. These results underline the role of PGI_2_ in the regulation of thrombus formation [136].

Clinically, these properties are used in the form of PGI_2_ mimetics (e.g., Epoprostenol, Iloprost, Beraprost, Trepostrinil, Selexipag, etc.) [137]. The most commonly used prostacyclin analogue in pulmonary arterial hypertension (PAH) is Epoprostenol [138]. Other formulations can be used as either IV or inhaled depending on the indication for treatment [139]. Analogues are more stable in vivo compared to the parent molecule and are applied, e.g., to treat patients suffering from PAH [140], critical limb ischemia, Bürger’s disease, Raynaud phenomenon and scleroderma diseases [141,142]. A comprehensive review of the complex and not yet fully elucidated mechanisms was provided recently by Lau and Lui [143]. A recently emerging new strategy in vascular diseases (except for PAH) is the local administration of PGI_2_ analogues to avoid the adverse effects of the systemic application [144].

### 3.5. Prostaglandin-D_2_ (PGD_2_)

Prostaglandin-D_2_ is well established as a macrophage (mast cell) product but, in lesser amounts, is also synthesized by platelets. It is a prostaglandin that binds and activates two distinct receptors: DP_1_ (via G_α(s)_ → AC) as well as DP_2_ [145,146]. It is rapidly metabolized enzymatically to 11-epi-prostaglandin-F_2α_ or 13,14 dihydro-15-keto-prostaglandin-D_2_ or non-enzymatically in aqueous solution to prostaglandin-J_2_ (PGJ_2_) [147]. The apparent half-life time in blood plasma is approximately 30 min, after which it loses its potential to inhibit platelet aggregation.

PGD_2_ is known to inhibit platelet aggregation [136], which follows the interaction with the DP_1_ receptor and AC activation [148]. The inhibitory effect is observed in human platelets—but not in murine platelets—due to the presence or absence of DP_1_ receptor coupling to G_s_.

Shuligoi et al. showed that incubation of plasma with PGD_2_ causes a time-dependent increase in the half maximal inhibitory concentration (IC_50_) for collagen-induced platelet aggregation (factor of 1.9% after 60 min and of 6.5% after 120 min) [149]. In this study, the PGD_2_ metabolite PGJ_2_ also inhibited collagen-induced platelet aggregation, although, 10- to 30-fold higher concentrations were required. Incubation of PGJ_2_ in plasma resulted in a very rapid decrease of its inhibitory potency. While the metabolites Δ12-PGJ_2_, 15d-PGJ_2_ and 15d-prostaglandin-D_2_ had no effect at concentrations up to 1 mM, Δ12-PGJ_2_ retained an inhibitory effect on collagen-induced platelet aggregation, which was comparable to PGJ_2_. The inhibitory potency of Δ12-PGJ_2_ was rapidly decreased by incubation in plasma.

In human platelets, the inhibitory potency of PGD_2_ was two-times higher than that of prostaglandin-E_1_, but much less than that of prostacyclin [125,136]. The therapeutic potential in cardiovascular diseases has not yet been studied in humans. During the 2000s and until today, several DP2 antagonists were studied in clinical trials for the treatment of asthma [150,151]. Antagonists of PGD_2_ receptor 2 have advanced into phase III clinical trials [152].

### 3.6. Prostaglandin-E_1_ (PGE_1_)

Prostaglandin-E_1_ is a product of the arachidonic acid metabolism in many cells and is—to some extent—generated by activated platelets [153,154,155]. It is largely metabolized during the first lung passage [156,157]. Of the resulting metabolites, the 13,14-dihydro derivate has an antiplatelet effect, like PGE_1_. The 15-keto-13,14-dihydro derivative has a considerably weaker effect [158]. Prostaglandin-E_1_ stimulates cAMP synthesis and inhibits platelet aggregation [159,160,161]. In human platelets, it can bind to IP—the PGI_2_ receptor—as well as to PGE receptors [162]. In mice, the rank order of affinity is EP3 > EP4 > EP2 > EP1 > IP [163]. However, the inhibitory effect of the molecule on human platelet aggregation can be blocked by an IP receptor antagonist but not by an EP4 receptor antagonist [164]. These data suggest that PGE_1_ inhibits platelet aggregation solely via the IP receptor route.

A synthetic analogue of PGE_1_, Alprostadil, is in clinical use as a vasodilator to prevent contrast-induced nephropathy or for patients with erectile dysfunction [165,166]. PGE_1_ has also been used for years to treat patients in advanced stages (stage III and IV) of peripheral arterial occlusive disease (see Cochrane Database Review) [167].

### 3.7. Prostaglandin-E_2_ (PGE_2_, Higher Concentrations)

In contrast to the response to low PGE_2_ concentrations, higher doses inhibit platelet activation. This inhibitory effect of PGE_2_ was significantly blunted but was not entirely abolished in murine platelets lacking IP receptors [168]. However, the affinity of the prostanoid for the human IP receptors appeared to be relatively low [72]. Studies by Smith, Iyú and Philipose indicated an inhibition of platelet aggregation (in mice and humans) by the EP2 and EP4 receptors [164,169,170]. Their data suggest that the reduced aggregation results from the selective activation of these receptors. It is noteworthy that the inhibitory potency of an EP4 receptor agonist was two rank orders higher than that of an EP2 receptor agonist and was as high as that of an IP receptor agonist in human platelets [168,171]. Studies in recombinant HEK 293 cells have shown that—at concentrations above 500 nmol/L—PGE_2_ can activate the DP1 receptor as well, leading to the above-mentioned cAMP-dependent inhibition of platelet function [172]. PGE_2_ is used in gynecology for labor induction (Dinoproston) but not as a platelet function inhibitor or in cardiovascular medicine [173,174].

### 3.8. Prostaglandin-E_3_ (PGE_3_)

This prostaglandin derives from omega-3 fatty acids and is synthesized by COX from eicosapentaenoic acid (EPA) [175,176]. It was reported to have anti-proliferative effects in different cancer cells [176], is involved in tumor angiogenesis [177] and can influence endothelial cell integrity [178,179]. A study by Iyu revealed a reduced platelet aggregation (PAF-induced) and expression of plasma membrane P-Selectin (U46619-induced) when platelets of human origin were treated with PGE_3_. These effects were concentration-dependent and enhanced when an EP3 receptor antagonist was applied additionally. In contrast, effects were inhibited when the EP4 receptor was antagonized, but were not influenced by an IP receptor antagonist. The overall influence of PGE_3_ on platelet function is consequently balanced by EP3 and EP4 receptor activation, which is in accordance with the PGE_2_ (EP3) but not the PGE_1_ (IP) receptor routes. These data indicate a potential mechanism of how omega-3 fatty acids—the precursors of PGE_3_—might influence platelet function [180,181,182]. At present, too little data is available on this aspect.

## 4. Conclusions

The understanding of the action of prostanoids and their receptors has led to the development of anti-platelet agents [183,184]. These are applied to prevent thrombotic events such as myocardial infarction or cerebral thrombosis [185], the major causes of death in developed countries [186]. The rank of order of potency of platelet activation inhibitors is: PGI_2_ > 6-keto-PGE_1_ > PGD_2_ > 6-keto-PGF_2α_ > PGE_2_ > PGF_2α_ [187]. The order reflects the importance of the prostacyclin receptor in mediating effects of prostaglandin on the platelet adenylyl cyclase. The targets of aspirin, prasugrel or cilostazol are cyclooxygenase, ADP receptor P2Y_12_ and phosphodiesterase. Although a PGI_2_ receptor agonist (PGI_2_ or PGE_1_ analogue) and a thromboxane synthase inhibitor have been used for anti-platelet therapy, there are still no anti-platelet agents targeting PGE receptors in clinical use. Previous studies revealed a role of the EP3 receptor in thromboembolism [63,70,188] and higher inhibitory potency of a EP4 agonist in platelet aggregation [168]. Altogether, the discussed data suggest a potential of respective antagonists and agonists as novel anti-platelet agents [169,189].

## Figures and Tables

**Table 1:** Prostanoid, receptor (sub) types and signaling pathways.

Prostanoid		PGD_2_		PGE_1_	PGE_2_				PGE_3_	PGF_2α_	PGI_2_	TXA_2_
Receptor		DP		IP, EP	EP					FP	IP	TP
	Subtype	DP_1_	DP_2_ CRTH_2_		(EP1)	EP2	EP3	EP4				TP_a_
G-protein	Linkage	G_s_	G_i_	G_s_	G_q_	G_s_	G_i_	G_s_		G_q_	G_s_	G_q_
Signaling pathway	AC	↑	↓	↑		↑	↓	↑			↑	
	Ca^2+^	↑	↑		↑		↑			↑		↑
	cAMP	↑	↓	↑		↑	↓	↑			↑	
	CREB				↑			↑				
	ERK				↑			↑				
	GSK3					↑						
	IP3				↑		↑			↑		↑
	PI_3_K							↑				
	p38 MAPK				↑			↑				
	PLC				↑		↑			↑		↑
	PKA							↑			↑	
	PKB (AKT)							↑				
	PKC				↑					↑		

↑: activation, increase, stimulation; ↓: inhibition, decrease. AC: adenylyl cyclase. Ca2+: calcium ion. cAMP: cyclic adenosine monophosphate. CREB: cAMP-response element-binding protein. ERK: extracellular signal-regulated kinases. GSK3: glycogen synthase kinase 3. IP3: inositol trisphosphate. PI3K: phosphatidylinositol 3-kinase. p38 MAPK: p38 mitogen-activated protein kinases. PLC: phospholipase-C. PKA: protein kinase A/cAMP-dependent protein kinase. PKB (AKT): activation of protein kinase-B. PKC: protein kinase-C. ( ): not shown in human platelets. Stimulatory effects of prostanoids on platelet aggregation.
